# Automatic identification of optimal marker genes for phenotypic and taxonomic groups of microorganisms

**DOI:** 10.1371/journal.pone.0195537

**Published:** 2018-05-02

**Authors:** Elad Segev, Zohar Pasternak, Tom Ben Sasson, Edouard Jurkevitch, Mira Gonen

**Affiliations:** 1 Department of Mathematics, Holon Institute of Technology, Holon, Israel; 2 Department of Plant Pathology and Microbiology, Faculty of Agriculture, Food and Environment, The Hebrew University of Jerusalem, Rehovot, Israel; 3 Department of Mathematics and Computer Science, The Open University of Israel, Raanana, Israel; 4 Department of Computer Science, Ariel University, Ariel, Israel; CPERI, GREECE

## Abstract

Finding optimal markers for microorganisms important in the medical, agricultural, environmental or ecological fields is of great importance. Thousands of complete microbial genomes now available allow us, for the first time, to exhaustively identify marker proteins for groups of microbial organisms. In this work, we model the biological task as the well-known mathematical “hitting set” problem, solving it based on both greedy and randomized approximation algorithms. We identify unique markers for 17 phenotypic and taxonomic microbial groups, including proteins related to the nitrite reductase enzyme as markers for the non-anammox nitrifying bacteria group, and two transcription regulation proteins, *nusG* and *yhiF*, as markers for the Archaea and *Escherichia/Shigella* taxonomic groups, respectively. Additionally, we identify marker proteins for three subtypes of pathogenic *E*. *coli*, which previously had no known optimal markers. Practically, depending on the completeness of the database this algorithm can be used for identification of marker genes for any microbial group, these marker genes may be prime candidates for the understanding of the genetic basis of the group's phenotype or to help discover novel functions which are uniquely shared among a group of microbes. We show that our method is both theoretically and practically efficient, while establishing an upper bound on its time complexity and approximation ratio; thus, it promises to remain efficient and permit the identification of marker proteins that are specific to phenotypic or taxonomic groups, even as more and more bacterial genomes are being sequenced.

## Introduction

The first complete bacterial genome sequence was published in 1995 [1]. Since then, sequencing technology has developed rapidly, causing a dramatic reduction in the cost of sequencing, which made bacterial genome sequencing affordable to a great number of labs [2]. The Ensembl database of whole genomes of bacteria has grown from about 9,000 to more than 40,000 in a few short years [3,4], and this number continues to increase exponentially each year [5]. This large number of bacterial genomes enabled us, for the first time, to identify marker genes for specific groups of microbial organisms based on the full complement of genes in each genome [6]. Currently, molecular typing of specific microbial groups is mostly done using either multi-locus sequence typing (MLST) or core genome MLST (cgMLST). In MLST, 5–16 (usually 7–8) housekeeping genes are selected as molecular markers and their sequence is compared between isolates [7]. However, the limited repertoire of highly conserved genes, as well as their sequence conservation, may sometime limit the discriminative power of this method, as evident in the typing of *Enterococcus faecium* [8]. The solution is usually to increase the number of genes, so in cgMLST, between 1500–3000 marker genes are used, which increases the discriminative power but forces any new isolate to be fully sequenced before it can be typed, thus requiring complex genomic analysis. A different way to improve MLST is by discarding the usage of housekeeping genes in favor of small groups of genes that are unique to specific taxonomic or phenotypic groups. This allows quick and affordable typing, using PCR instead of whole-genome sequencing, while retaining high discriminative power.

When using non-housekeeping marker genes for the MLST scheme, choosing the right ones is critical: non-representative or non-unique genes can lead to erroneous typing [8], or to an inefficient process due to requiring too many marker genes per group in order to verify the genomes' membership. Genetic markers are often selected ad hoc, using too few reference genomes and/or manual inspection of the results [9]. Therefore, an algorithm for finding optimal markers for specific groups of organisms is of great value to ecological and medical research [10,11]. Several software tools were developed for this purpose, but these are mostly limited to a single pathogenic organism [12], are computationally intensive [13], or can only identify marker genes for defined taxonomic groups [14,15]. Existing methods are not capable of creating novel typing schemes for any group of genomes (from one to thousands) by user choice, in a user-friendly manner while operating quickly and efficiently on any personal computer. We use an innovative approach for this problem, mapping it using the well-known Hitting Set (HS) mathematical problem. To increase the discriminative power of the approach, we use polypeptides instead of genes. Given a set of bacteria where each bacterium is represented by the set of the proteins it contains, a subset of bacteria to type will contain a minimum subset of specific proteins not found in the other bacteria. This minimum subset of proteins is thus present in every bacterium of the subset, marking the phenotype or taxon, while at least one protein is missing in the other bacteria, which do not have the same phenotype or do not belong to the taxon. The problem of typing a set of bacteria can thus be solved mathematically.

To solve this problem, we start from the set of all of the proteins that are present in all the bacteria in our database. These proteins will be marked as *P*_1_, *P*_2_ etc. in our case; a bacterium is defined as the set of proteins that it consists of.

**Fig 1:**
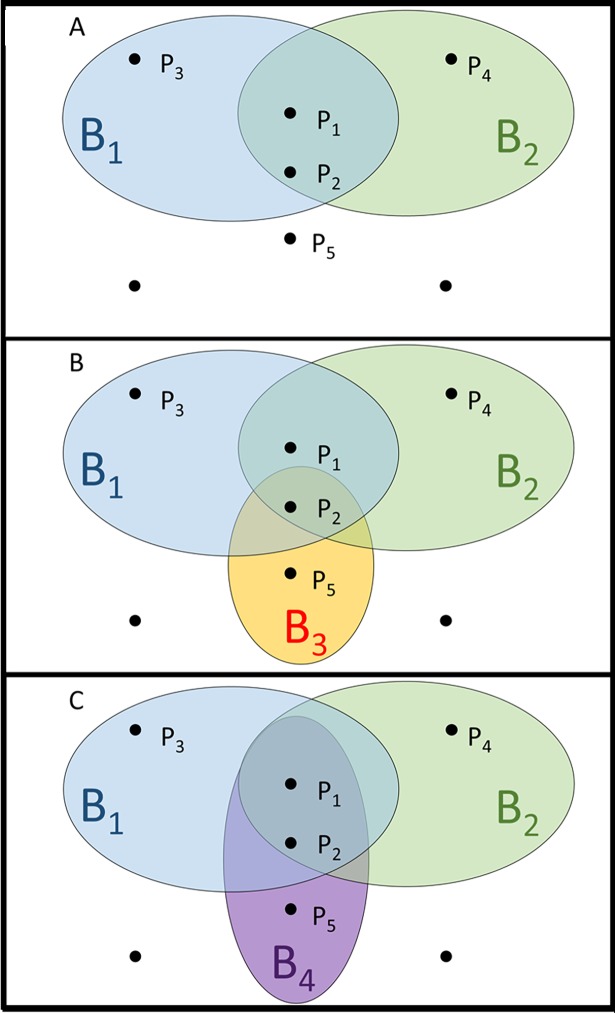
Graphical representation of the proteins (denoted P_1_, P_2_, P_3_, P_4_, P_5_) which can serve as markers for the bacterial (denoted B_1_, B_2_, B_3_, B_4_) group of interest consisting of B_1_ and B_2_: (A) shows that P_1_, P_2_ can serve as a minimal set of markers for the group of interest; (B) P_1_ only can serve as a marker for the group of interest; and (C) there are no markers for the group of interest.

For example, as seen in Fig 1A, bacterium no. 1 (denoted B_1_) is defined as the set of *P*_1_, *P*_2_ and *P*_3_, meaning that this bacterium contains only these three proteins, and bacterium B_2_ contains only proteins P_1_, P_2_ and P_4_. Given that B_1_ and B_2_ form a group of interest which we wish to identify, P_1_ or P_2_ can serve as marker proteins for this group of interest. Thus, the minimal set of proteins which identify this group can be either P_1_ or P_2_. If a third bacterium exists, which is not included in the group of interest and consists of P_2_ and P_5_, then P_2_ loses its identifying property for the group of interest and the minimal set becomes P_1_ only (Fig 1B). Not every group of interest can be exclusively identified by a set of proteins: in some cases, such as Fig 1C, where B_4_ is not a part of the group of interest and contains P_1_, P_2_ and P_5_, there is no set of proteins that can identify the *B*_1_ ∪ *B*_2_ group. As the number of proteins and bacteria increases, an exact solution for finding a minimal set of proteins out of millions of known proteins in order to identify any group of interest out of thousands of organisms becomes impossible to solve in a reasonable timeframe. We will show that this problem cannot be solved efficiently since it is NP-hard [16,17], namely, no efficient algorithm for solving this problem is known. The definition of the hitting set problem is the following: given a ground set *S* and a collection *C* of subsets of *S*, find a hitting set with a minimum cardinality, i.e., a subset *S*′ ⊆ *S* such that *S*′ contains at least one element from each subset in *C*. We will elaborate on the exact connection between the hitting set problem and our problem of identifying a set of proteins in the Materials and Methods subsection named Problem Definitions and Notations. In S1 Algorithm, we show that even if we are willing to relax our problem to that of finding a hitting set of a limited size, an exact approach is impractical. Since this problem is of great importance, a lot of effort has been made to find efficient approximation algorithms to it. That is, efficient algorithms that return a solution to the hitting set problem, which is at most *r* times the size of a minimum set, where *r* is the approximation factor. Here, we apply an approximation algorithm which finds relatively small sets of proteins that identify the group of interest. Obviously, these sets are not necessarily minimal. For example, in Fig 1A, an approximate solution might be the set containing both P_1_ and P_2_, whereas an exact (i.e. minimal) solution will be either P_1_ or P_2_.

To find an approximation algorithm to the hitting set problem, we explore approximation algorithms to an equivalent problem, the “set cover” problem [16], which in addition to several known approximation algorithms has many heuristics for solving it efficiently [18–27]. The definition of the set cover problem is the following: given a ground set *S* and a collection *C* of subsets of *S*, find a subset *C*′ ⊆ *C* such that every element in *S* is contained in some subset in *C*′. When considering the most suitable algorithm for our work, we looked for algorithms that will be both theoretically and practically efficient. It was proven that the set cover problem is approximable within *O*(*log*|S|), where |*S*| is the size of the ground set S, using a greedy algorithm [28–31]. Therefore, a greedy algorithm can achieve the same corresponding approximation ratio as the hitting set problem. Moreover, a randomized algorithm based on linear programming achieves the same approximation ratio with a probability of at least 1-1/|*S*| [32]. It was shown that the set cover problem cannot be approximated in polynomial time to within a factor of (1 − *o*(1)) · *ln*|*S*| (unless *P* = *NP*) [33,34]. This means that the greedy approximation algorithm and the randomized algorithm theoretically achieve the best possible ratio. Thus, we implemented the greedy algorithm and a randomized algorithm based on randomized rounding of linear program constraints [35–38]. Both algorithms were used to identify non-anammox nitrifying bacteria and predatory bacteria as phenotypic groups, Archaea and *Escherichia/Shigella* as taxonomic groups, and 13 different pathogenic sub-groups of *E*. *coli* as combined phenotypic/taxonomic groups.

## Materials and methods

*Biological Data*. Genome and protein data were obtained as outlined in [6] from the 2016 version of the orthologous protein cluster table created and maintained by the microbial genome database (MBGD) [39] and freely available at http://mbgd.genome.ad.jp/htbin/view_arch.cgi. This table is updated yearly and is arranged so that each row is an orthologous cluster (i.e. the same protein) and each column is a genome. Ortholog identification and grouping is performed by the DomClust [40] and DomRefine [39] procedures, with MergeTree [41] adding new genomes to the table. Ortholog classification is based on all-against-all clustering with local alignment of the protein domain sequences. The raw MBGD data were automatically cleaned, as outlined in [6]: unnecessary and redundant data were deleted, and all protein occurrences were transformed from protein names into binary data so that each datapoint in the table contains either a one or a zero only (the protein is present or absent in the genome, respectively). This reduces the file size by two orders of magnitude and enables efficient algorithm usage. This approach enables one to make use of any orthologous group classification, for example splitting or merging groups according to the biological system. In order to fully challenge our methods and algorithms, we implemented the algorithm using 17 different microbial groups which represent a wide variety of classification criteria: non-anammox nitrifying bacteria and predatory bacteria as phenotypic groups, Archaea and *Escherichia/Shigella* as taxonomic Groups, and 13 different subtypes of pathogenic *E*. *coli* as taxonomic/phenotypic groups. First, of the 4742 complete (i.e. non-draft) genomes of bacteria and Archaea available in the MBGD database, 12 belonged to non-anammox nitrifying bacteria from two groups: ammonia oxidizers (*Nitrosomonas europaea* ATCC 19718, *Nitrosomonas eutropha* C71, *Nitrosomonas* sp. AL212, *Nitrosomonas* sp. Is79A3, *Nitrosospira multiformis* ATCC 25196, *Nitrosococcus halophilus* Nc4, *Nitrosococcus oceani* ATCC 19707, *Nitrosococcus watsoni* C-113, *Nitrobacter hamburgensis* X14, *Nitrosomonas communis* Nm2) and nitrite oxidizers (*Nitrobacter winogradskyi* Nb-255, *Nitrospira defluvii*). Genomes of bacterial nitrifiers using anaerobic ammonium oxidation (anammox) were not available. Second, MBGD contained 16 genomes belonging to known predatory bacteria: *Bdellovibrio bacteriovorus* HD100, *Bdellovibrio bacteriovorus* 109J, *Bdellovibrio bacteriovorus* tiberius, *Bdellovibrio bacteriovorus* W, *Bdellovibrio exovorus*, *Bacteriovorax marinus* SJ, *Cytophaga hutchinsonii* ATCC 33406, *Flavobacterium johnsoniae* UW10, *Herpetosiphon aurantiacus* ATCC 23779, *Micavibrio aeruginosavorus* ARL-13, *Micavibrio aeruginosavorus* EPB, *Myxococcus xanthus* DK 1622, *Sorangium cellulosum* So ce 56, *Sorangium cellulosum* So0157-2, *Saprospira grandis* Lewin, *Stigmatella aurantiaca* DW4/3-1. Third, we obtained all 226 archaeal genomes available at MBGD, comprising all archaeal classes and families (Table A in S1 File); fourth, the 147 genomes belonging to the *Escherichia/Shigella* bacterial genus (Table B in S1 File); and fifth, the genomes comprising 13 *E*. *coli* pathotypes (Table B in S1 File). The taxonomic and phenotypic identifications were based on the latest published data[42]. E. coli strain pathogroup assignment was according to the EnteroBase database freely available at https://enterobase.warwick.ac.uk

*Problem Definition and Notation*. Given a set B of bacteria, a subset *B* of B, and a set of orthologous proteins *P*, we want to find a minimum subset $\hat{P}$ of *P* that would identify the bacteria in *B*. Namely, all orthologous proteins in $\hat{P}$ are in every bacterium in *B*, and for every bacterium in $\bar{B}≔B\backslash B$ there is at least one ortholog protein in $\hat{P}$ that is missing. We consider each bacterium as a subset of the orthologous proteins it contains. Thus, the formal definition of the problem is as follows: given a ground set of m elements *P* = {*p*_1_…,*p*_*m*_}, a collection B of *n* subsets $B=\{B_{1},\ldots ,B_{n}\}$, such that *B*_*j*_ ⊆ *P* and |*B*_*j*_| ≥ 1 for all 1 ≤ *j* ≤ *n*, and a subcollection B⊆B of size *k*, *B* = {*B*_1_…,*B*_*k*_}, we want to find a minimum size subset of *P*, $\hat{P}$, such that for each $p\in \hat{P}$ it holds that $p\in \overset{k}{\underset{j=1}{⋂}}B_{j}$, and for each $B_{j}\in B\backslash B$ it holds that there exists some $p\in \hat{P}$ such that *p* ∉ *B*_*j*_. *Algorithm Development*. We use a greedy approximation algorithm and a randomized approximation that is based on Linear Programming for the hitting set problem. An advantage for the random algorithm is that different runs of the algorithm may produce different results in an efficient manner.

### Algorithm 1, based on hitting set

1. $\hat{P}\leftarrow B_{1}.$
2. *for all $p\in \hat{P}$* *do*
3. *if p ∉ B_2_ ∩ B_3_ ∩…∩ B_k_ then* *$\hat{P}\leftarrow \hat{P}\backslash \{p\}$*
4. *for k + 1 ≤ j ≤ n let $\tilde{B_{j}}=\{p\in \hat{P}|p\notin B_{j}\}$*.
5. *let $\tilde{B}=\{{\tilde{B}}_{k+1},\ldots ,{\tilde{B}}_{n}\}$*.
6. *if $\tilde{B}$ contains an empty set–return "no hitting set"*.
7. *run an algorithm of the hitting set problem on the input $\hat{P},\tilde{B}.$ Namely, sub algorithm 1 or 2 on input $\hat{P},\tilde{B}$, respectively*.

We can conclude that there is no possible hitting set for an instance of a problem in step 5 even before running an explicit sub-algorithm for the hitting set problem. Let $\hat{P}$ and $\tilde{B}$ be the result sets at the end of stage 4 in algorithm 1. If at least one of the sets in $\tilde{B}$ is empty, then obviously there is no hitting set. Notice that if all sets in $\tilde{B}$ are not empty, there is always a hitting set since we can take $\hat{P}$. By the construction of sets in $\tilde{B},\;\hat{P}$ must hit each set $B_{i}\in \tilde{B}$ if it is not empty. As noted in the previous subsection, the hitting set problem is equivalent to the set cover problem. Moreover, we can use any algorithm to the set cover problem to solve the hitting set problem. Consider an instance *S*, *C* to the set cover problem. We define the following instance to the hitting set problem. The ground set is defined to be $\hat{S}$ = C, namely, each element is a subset of the instance to the set cover problem. For each element in *e* ∈ *S*, we define a subset of $\hat{S}$ which is the set of subsets of *S* that contain *e*. Therefore, a minimum cardinality cover of *S*, *C* is a minimum cardinality hitting set of $\hat{S},\hat{C}$. To solve Item 6 of Algorithm 1 we first use the following greedy algorithm for the hitting set problem:

### Sub algorithm 1, greedy algorithm for hitting set (S, C)

*Input: universe S = {s_1_,…,s_m_}, C = {C_1_,…,C_n_}, s.t. C_i_ ⊆ S for all 1 ≤ i ≤ n*.

*Output: $\hat{S}\subseteq S$*.

1. $\hat{S}\leftarrow \emptyset .$
2. $\hat{C}\leftarrow C$.
3. *while $\hat{C}\neq \emptyset$ do*
  - *Select s ∈ S such that s hits the largest number of subsets in* *$\hat{C}$ (i.e. select s s.t. $\left|\left\{C_{i}\in \hat{C}|C_{i}\cap \{s\}\neq \emptyset \right\}\right|$ is of maximum cardinality)*
  - *Remove the hit subsets from $\hat{C}$, namely $\hat{C}\leftarrow \hat{C}\backslash \{C_{i}\in \hat{C}|C_{i}\cap \{s\}\neq \emptyset \}$*.
  - $\hat{S}\leftarrow \hat{S}\cup \{s\}$.
4. *Return $\hat{S}$*.

**Lemma 1** given an instance (S,C), sub-algorithm 1 finds a hitting set of size of at most *OPT* ⋅ *O*(log|*C*|) = *OPT* ⋅ *O*(log *n*), where OPT is the size of an optimal HS, with time complexity *O*(min{*m*,*n*} ⋅ *n* ⋅ *m*).

**Proof** The correctness and approximation ratio of the lemma follows from the correctness and approximation ratio of the greedy algorithm for the set cover problem [29], and the tight connection between the set cover problem and the hitting set problem [43]. To compute the time complexity of the algorithm, let ${\hat{C}}_{j}$ be the set that needs to be hit at step *j*, and let ${\hat{S}}_{j}$ be the set of elements that have not been selected yet at step *j*. The most expensive operation in the loop of Item 3 is Item 3a. In the worst case of Item 3a for every *s* ∈ *S*, the algorithm goes over all the subsets $C_{i}\in {\hat{C}}_{j}$ to check whether *s* ∈ *C*_*i*_. Using a reasonable data structure, we can assume that the access time for checking whether *s* ∈ *C*_*i*_ is *O*(1). Therefore, the running time of Item 3a at step *j* is $\left|{\hat{S}}_{j}|\cdot (\sum_{C_{i}\in {\hat{C}}_{j}}O(1))=|{\hat{S}}_{j}|\cdot |{\hat{C}}_{j}\right|$. Let l be the maximum number of steps the loop in Item 3 is performed. Obviously l≤min{m,n}. Thus, time complexity of Sub-Algorithm 1 is.

$$O\left(\sum_{j=1}^{l}\left|{\hat{S}}_{j}|\cdot |{\hat{C}}_{j}\right|\right)=O\left(\sum_{j=1}^{\min\left\{m,n\right\}}\left(m-j)\cdot (n-j\right)\right)=O(\min\left\{m,n\right\}\cdot n\cdot m)$$

Notice that the theoretical upper bound on the time complexity can be reduced for specific instances of the problem. For example, if every element in *S* appears in many subsets in *C* then the number of steps l, of performing the loop in Item 3 of Sub-Algorithm 1 is much smaller than n.

Lemma 1 implies the following theorem:

**Theorem 1** Algorithm 1 finds a set of proteins $\hat{P}$ such that $\left|\hat{P}|=O(log(n-k)\cdot |P_{OPT}|\right)$, where *P*_*OPT*_ is an optimal set of proteins that identifies *B*, with time complexity of *O*(*m*^2^ ⋅ *n*).

**Proof**: We first note that the initial set $\hat{P}$ is an intersection of all the subsets of proteins in the tested set {*B*_1_,…,*B*_*k*_}. Therefore, any set returned by the algorithm to identify the tested set must be a subset of the initial $\hat{P}$ Moreover, the returned set must not identify the control set {*B*_*k+*1_,…,*B*_*n*_}, Since Sub-Algorithm 1 returns a subset of $\hat{P}$ that is a hitting set of $\tilde{B}$, for each bacteria *B*_*j*_ of the control set {*B*_*k+*1_,…,*B*_*n*_} the returned $\hat{P}$ must include at least one protein that does not exist in *B*_*j*_. This implies the correctness of the algorithm.

By lemma 1, with *S* = *B*_*1*_ ∩…∩ *B*_*k*_ and $C=\tilde{B}$, it holds that $|\hat{P}|=OPT\cdot O(\log|\tilde{B}|)=OPT\cdot O(\log\left(n-k)\right)$, where *OPT* is an optimal solution to the HS problem on the given *S*, *C*. Notice that by the definition of our problem, every optimal solution for identifying *B* needs to find a HS of $\tilde{B}$ as well. Thus, it holds that *OPT* = |*P*_*OPT*_|, so $\left|\hat{P}|=|P_{OPT}|\cdot O(log(n-k)\right)$, as claimed.

To prove the bound on the time complexity, it must be noted that the running time of Item 2 of Algorithm 1 is $O(|B_{1}|\cdot \sum_{j=1}^{k}\left|B_{j}\right|)$. The running time of Item 3 of Algorithm 1 is

$$O\left(\left|\hat{P}\right|\cdot \sum_{j=k+1}^{n}\left|B_{j}\right|\right)=O\left(\left|B_{1}\cap \ldots \cap B_{k}\right|\cdot \sum_{j=k+1}^{n}\left|B_{j}\right|\right)=O\left(\left|B_{1}\right|\cdot \sum_{j=k+1}^{n}\left|B_{j}\right|\right).$$

By Lemma 1 the time complexity of finding the hitting set in item 5 on $\left(\hat{P},\tilde{B}\right)$ is

$$O(\min\left\{\left|B_{1}\cap \ldots \cap B_{k}|,(n-k\right)\right\}\cdot |B_{1}\cap \ldots \cap B_{k}|\cdot (n-k))=O(\min\left\{\left|B_{1}|,(n-k\right)\right\}\cdot |B_{1})\cdot (n-k)).$$

Therefore, the total time complexity of the algorithm is

$$O\left(\left|B_{1}\right|\cdot \sum_{j=1}^{n}|B_{j}|+\min\left\{|B_{1}|,n-k\right\}\cdot |B_{1}|\cdot (n-k)\right)=O(m^{2}\cdot n+\min\left\{m,n\right)\cdot m\cdot (n-k)=O(m^{2}\cdot n).$$

This completes the theorem.

Notice that again, in practice, the actual time complexity is smaller than our upper bound, which makes our algorithm very fast in reality, as demonstrated in the next section. We now show another version of Algorithm 1 in which we replace Sub-Algorithm 1 by a Linear Programming randomized algorithm for the hitting set problem. For 1 ≤ *i* ≤ *m* set *x*_*i*_ = 1 if $s_{i}\in \hat{S}$ and *x*_*i*_ = 0 otherwise. Thus, finding a hitting set can be formulated as an integer linear program. We relax the integer linear program to a fractional one, and then use randomized rounding to get an integer solution.

### Sub Algorithm 2, linear programming randomized algorithm for hitting set (S, C)

*Input: universe S = {s_1_,…,s_m_}, C = {C_1_,…,C_n_}, s.t. C_i_ ⊆ S for all 1 ≤ i ≤ n*.

*Output: $\hat{S}\subseteq S$*.

1. Solve the following fractional linear programming problem:
  $$Minimize\;\sum_{i=1}^{m}x_{i}$$
  $$subject\;to\;\sum_{i:s_{i}\in C_{j}}x_{i}\geq 1,\;\forall C_{j}\in C$$
  $$0\leq x_{i}\leq 1,1\leq i\leq m$$
2. *For all 1 ≤ i ≤ m let ${\hat{x}}_{i}$ be the value assigned to x_i_, in an optimal fractional solution of the previous linear program*.
3. *Let X_i_, 1 ≤ i ≤ m be independent random variables such that*
  $$Pr\left[X_{i}=0\right]={\left(1-{\hat{x}}_{i}\right)}^{c\cdot log\;n},for\;constant\;c,\;and$$
  $$Pr\left[X_{i}=1\right]=1-{\left(1-{\hat{x}}_{i}\right)}^{c\cdot \log n}.$$
4. $\hat{S}\leftarrow \{s_{i}\in S|X_{i}=1\}.$
5. *Return $\hat{S}$*.

According to [35–38] Sub-Algorithm 2 returns a hitting set of size *c* ⋅ log *n* times the size of an optimal solution, with a probability of at least $1-\frac{1}{2^{c}}$. Therefore Algorithm 1, when using Sub-Algorithm 2, achieves, with high probability, the same theoretical approximation ratio of Algorithm 1 when using Sub-Algorithm 1. As the constant *c* presented in item 3 of the algorithm grows, the probability that the result is indeed a valid HS grows, but the expected size of the result also increases. Based on our experience, choosing the constant *c* = 1 gives us a relatively small error probability, while not increasing the received HS size. In each case the random algorithm returned a HS, we verified that it is indeed a HS. The time complexity of Sub-Algorithm 2 is derived mainly from the time complexity of solving the Linear Program of the problem in step 1. We have used Dantzig's Simplex method [44] to solve linear programs. This method has an exponential time complexity in the worst case but is highly efficient in practice. It is possible to rerun this sub algorithm efficiently as we note that once we have solved the linear program, we are left with assigning a binary value for each variable and then deciding whether the result is an actual hitting set, so the time complexity of a single rerun for an instance of this algorithm is *n* ⋅ *m*. For our purposes, we have found that 10,000 reruns of this algorithm per instance produces reasonably low runtime and at the same time produces a large variety of interesting outcomes. In some occasions, the result of the randomized algorithm may be optimal and deterministic. If the solution for the linear program is binary, that is, each variable receives either 0 or 1, then we have found a hitting set without the need for linear relaxation. That is the optimum value for the integer program of the given instance for exact hitting set program. In that case, it is redundant to rerun the algorithm as it will generate the same optimal result. This randomized approach presents several different hitting sets.

**Fig 2:**
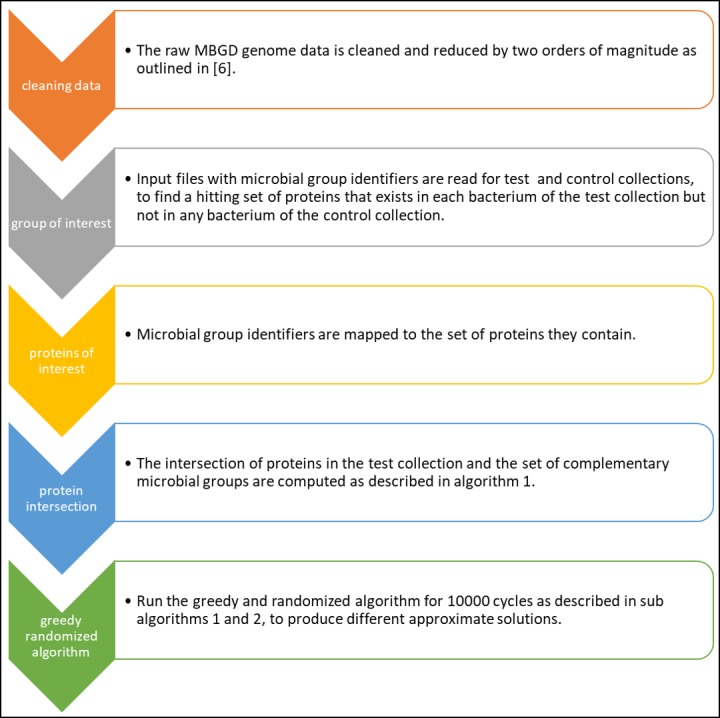
Software structure and output.

An overview representation of the software structure is presented in Fig 2. This section details the description of the algorithm. The source code can be downloaded directly from https://www.dropbox.com/sh/s6u8fh69ygzkuuk/AABLpFPyWjY3kLGID6H2cG-Ja?dl=0

The algorithm may produce three types of solutions: (i) Optimal minimal solutions. It is sometimes possible to guarantee for a result set to be minimal (rather than an approximation) as a result of a possible optimal solution for the linear program for the HS formulation with sub-algorithm 2. (ii) Collection of approximated solutions. One or more hitting sets that are no larger than *OPT* ⋅ *O*(log *n*) where *OPT* is the minimal solution and *n* is the number of sets to hit (microbial groups in our case). (iii) No solution. In the process of algorithm 1, it is possible to say whether there is no set that exclusively hits all of the tested groups of an instance of the problem.

## Results and discussion

Our algorithms were employed to find the hitting set of 17 different microbial groups representing a wide variety of classification criteria (Table 1): 1) The non-anammox nitrifying bacteria, a group distinct from all other bacteria by its metabolic phenotype; 2) The second phenotypic group was the predatory bacteria, a group distinct by its trophic phenotype; 3) The third tested group was a taxonomic one–Archaea, a group distinct from Bacteria by domain-level taxonomy; 4) The fourth tested group was a taxonomic one: the *Escherichia/Shigella* groups, distinct by genus-level taxonomy, and; 5) The other 13 tested groups were of pathogenic *E*. *coli*. each distinguished from all other bacteria by both taxonomy and phenotype. In all cases where hitting sets were found, one of the hitting sets found by the random algorithm was identical to the hitting sets found by the greedy algorithm. The non-anammox nitrifiers used in this study are a group of 12 bacteria with a known genome, which perform the nitrification of ammonia or ammonium to nitrite. Five minimal HS were discovered, each containing five proteins (Table C in S1 File). Among these proteins were nitrite reductase (NirK)[45], an enzyme that catalyzes the reduction of nitrite to nitric oxide; siroheme synthase (CysG), an enzyme involved in the synthesis of siroheme, a heme-like prosthetic group used by the nitrite reductase [46]; Formate/nitrite transporter (NirC), a transmembrane channel that transports nitrite in and out of the cell [44]; and a nitric oxide reductase activation protein (NorD)[45], involved in reducing nitric oxide to nitrous oxide. Although it may appear that these proteins are only relevant to the first group of non-anammox nitrifiers (i.e. nitrite oxidizers), it was actually discovered that the second group (ammonia oxidizers) also depend on the nitrite reductase enzyme for efficient growth by its oxidation of ammonia to nitrite via hydroxylamine [47]. Thus, many of the HS proteins that our algorithm discovered are highly relevant to the biological phenotype that is unique to all non-anammox nitrifying bacteria, serving as a kind of "positive control" for our algorithm. The second phenotypic group, predatory bacteria, was not found to have any hitting sets proteins when compared to other (i.e. non-predatory) bacteria. This confirms previous studies (e.g. Pasternak et al. [48]), which also concluded that bacterial predation is not facilitated by unique proteins, and serves as a kind of "negative control" for our algorithm. In many examples bacteria performing similar metabolic functions (e.g. nitrification) all use similar enzymes to carry out the process. However, predation is functionally a more diverse process, and there are no molecular signatures specific to bacterial predation. Indeed, several features were shown to be highly enriched in predators, including adhesins, proteases and particular metabolic proteins, used for binding to, processing and consuming prey, respectively; in addition, most predators use the mevalonate pathway of isoprenoid biosynthesis, whereas almost all other bacteria use the DOXP pathway [48]. However, all of these proteins are also found in the genomes of non-predatory bacteria, and the differences might not lie in their presence or absence but rather in transcriptional and/or post-transcriptional regulation.

**Table 1:** Hitting sets (marker proteins) of 17 microorganism groups. HS, hitting set. Min., minimal. Greedy and random refer to the algorithm type. Phen., phenotypic. Tax., taxonomic. AIEC, adherent-invasive *E*. *coli*. EPEC, enteropathogenic *E*. *coli*. UPEC, uropathogenic *E*. *coli*. STEC, Shiga toxin-producing *E*. *coli*. NMEC, neonatal meningitis-associated *E*. *coli*. ExPEC, extra-intestinal pathogenic *E*. *coli*. ETEC, enterotoxigenic *E*. *coli*. EIEC, enteroinvasive *E*. *coli*. EHEC, enterohemorrhagic *E*. *coli*. EAEC, enteroaggregative *E*. *coli*. APEC, avian pathogenic *E*. *coli*. EAHEC, enteroaggregative hemorrhagic *E*. *coli*.

Group name	Group distinction	Run time (sec)	No. of HS genes (greedy)	Min. no. of HS genes (random) with representative genes	No. of HS with min. no. of HS genes (random)	Proven optimal HS
Nitrifying bacteria	Phen.	7.32	5	5 (NirK, NirC)	5	0
Predatory bacteria	Phen.	7.11	-	-	0	-
Archaea	Tax. (domain)	7.28	1	1 (NusG)	1	1
*Escherichia/Shigella*	Tax. (genus)	7.38	2	2 (YhiF)	10	0
All pathogenic *E*. *coli*	Phen. and tax.	7.35	-	-	0	-
AIEC	Phen. and tax.	7.35	3	3 (TnpR)	9	0
EPEC	Phen. and tax.	7.73	5	5 (YedK, ImpC)	7	0
UPEC	Phen. and tax.	7.39	-	-	0	-
STEC	Phen. and tax.	7.36	-	-	0	-
NMEC	Phen. and tax.	7.31	-	-	0	-
ExPEC	Phen. and tax.	7.37	-	-	0	-
ETEC	Phen. and tax.	7.37	-	-	0	-
EIEC	Phen. and tax.	7.43	-	-	0	-
EHEC	Phen. and tax.	7.30	-	-	0	-
EAEC	Phen. and tax.	7.34	-	-	0	-
APEC	Phen. and tax.	7.37	-	-	0	-
EAHEC	Phen. and tax.	7.38	4	3 (FliC)	1	1

**Fig 3:**
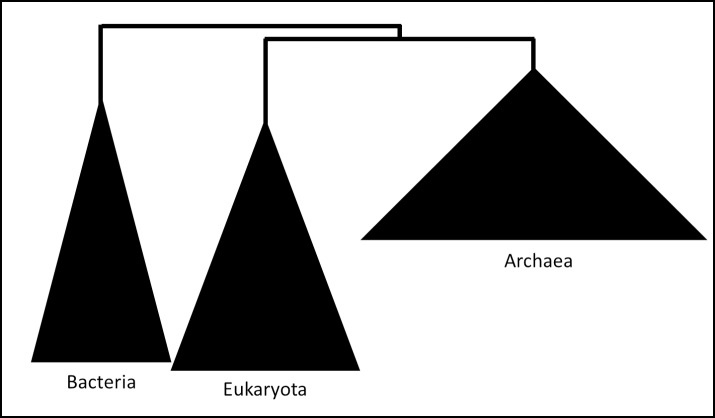
Maximum-likelihood phylogenetic tree of the NusG protein. All archaeal NusG sequences were taken from the GenBank database, along with their most similar bacterial and eukaryotic homologs for a total of 500 protein sequences. The bootstrap consensus tree inferred from 100 replicates was taken to represent the evolutionary history of the taxa analyzed. Branches were merged at the domain level.

The next group, Archaea, was compared to all the bacterial genomes in the database and an optimal single protein (NusG) HS solution was found to sufficiently distinguish the groups. NusG is a transcription elongation factor that binds to RNA polymerases and assists in RNA synthesis from a DNA template[49]. RNA polymerases are under strong evolutionary pressure to maintain their structure; therefore, the structure and sequence of NusG should also be conserved. In fact, this protein is considered to be the only transcription elongation factor whose sequence is universally conserved in all three domains of life: Bacteria, Archaea and Eukarya [50]. Surprisingly, our analysis found the amino-acid sequence to be very distinct between Archaea and the other two domains, and a consequent phylogenetic analysis of 500 NusG protein sequences (Fig 3) clearly confirmed this difference. Since this solution is an optimal solution the algorithms are deterministic—meaning there might be other genes specifically conserved only in Archaea in the current database which the algorithm ignores.

The fourth tested group is a taxonomic one; the bacterial genus *Escherichia/Shigella* was characterized by ten hitting sets, all with two proteins, where one of them is the YhiF protein which is a transcription regulator (Table D in S1 File). YhiF takes part in modulating levels of expression of LEE (locus of enterocyte effacement) proteins—a group of proteins responsible for attaching and effacing to the intestine of the host. Through the mechanisms of control of LEE expression, YhiF appears to play a central role in *Escherichia/Shigella* colonizing the host intestinal epithelium [51]. However, as it is present in all *Escherichia/Shigella* strains, including commensal ones, it is a taxonomic marker rather than a pathogenicity marker.

Finally, we searched for hitting sets which define 13 different subgroups—or pathotypes—of pathogenic *E*. *coli* bacteria. These pathotypes are defined according to their clinical symptoms (e.g. enterohemorrhagic) and therefore most do not have perfect marker proteins, i.e. ones that exist in all the strains of a specific pathotype and only in those strains [49]. Indeed, our analysis found that 10 of these 13 pathogroups showed no hitting sets, confirming that genomes within each group do not necessarily have taxonomic or phenotypic affiliation with each other and therefore non-housekeeping marker proteins are impossible to find in most groups. Even so, three important pathotypes could be efficiently defined: adherent-invasive *E*. *coli* (AIEC) with minimal hitting sets containing three proteins (Table E in S1 File), enteropathogenic *E*. *coli* (EPEC) with five proteins (Table F in S1 File) and enteroaggregative hemorrhagic *E*. *coli* (EAHEC) with three proteins (Table G in S1 File). EPEC is an important cause of diarrhea and premature death in children, especially in developing countries [51]; currently, the major diagnostic marker for EPEC is the *eae* gene (coding for intimin, an outer membrane adhesive protein), yet genomes from other pathotypes also possess this gene [52], making it a non-optimal marker. AIEC is not associated with diarrhea but is thought to contribute to the development of Crohn’s disease, a chronic inflammatory bowel syndrome [53]. It currently has no known diagnostic markers [52]. EAHEC is associated with food poisoning in the developed world [54]. Many of the hitting sets proteins that we found are either uncharacterized (i.e. 'hypothetical') or are from a viral (bacteriophage) source with DNA cut-and-paste functionality, e.g. transposases and integrases. One of our EAHEC HS members, FliC, has been used before as a marker for this group [54]. *E*. *coli* subtyping schemes are invaluable in identifying outbreaks and treating infection patients, but the current subtyping technology is imprecise and potentially misleading because *E*. *coli* genomes constantly change and evolve.

Our algorithm enables the accurate identification of marker genes for any microbial group, depending on the completeness of the database. Once marker genes are established and confirmed, new and unknown genomes can quickly be assigned to their group via MLST, without the need for whole-genome sequencing. specifically, our study found potential marker genes which in the future may enable reliable diagnosis of the EAHEC, EPEC and AIEC strains of pathogenic *E*. *coli*, thus improving the treatment of *E*. *coli-*related diseases. In addition to microbial identification, such analysis may help uncover novel genes pertinent to the grouping, such as virulence-associated or habitat-specific genes. As these genes are group-specific, they are prime candidates for further research which aims to understand the genetic basis of the group's phenotype as well as possible targets for antibiotic treatment. Finally, our algorithm may also help discover novel functions which are uniquely shared among a group of microbes. For example, several uncharacterized ("hypothetical") genes were found in this study to be pathogroup-specific; further investigation of these genes and proteins may reveal their possible function in connection to their specific group, leading to improved understanding and specific antibacterial treatments.

## Supporting information

S1 File Microbial groups and their genomes, and hitting sets (HS) found in this study. Table A in S1 Fie) Archaea genomes; Table B in S1 File) *Escherichia*/*Shigella* genomes; Table C in S1 File) nitrifiers HS; Table D in S1 File) *Escherichia*/*Shigella* HS; Table E in S1 File) AIEC (adherent-invasive *E*. *coli*) HS; Table F in S1 File) EPEC (enteropathogenic *E*. *coli*) HS. Table G in S1 File) EAHEC (enteroaggregative hemorrhagic *E*. *coli*) HS.
(XLSX)

S1 Algorithm A mathematical explanation for the necessity of approximation solution. (DOCX)
