# Supplementary material for: Automatic identification of optimal marker genes for phenotypic and taxonomic groups of microorganisms
Source: PLoS One. 2018 May 2;13(5):e0195537. doi: 10.1371/journal.pone.0195537 (PMC5931505; doi:10.1371/journal.pone.0195537)
Supplement: S1 Algorithm — (DOCX) [file pone.0195537.s002.docx]

**Supporting Information** **S1 algorithm:**

Motivation for approximate solution.

A different non-approximate approach for the problem might seek to ease the question at hand by limiting the size of the result hitting set. That is, we introduce a new constant $k$ and the algorithm would produce a minimal hitting set of size at most $k$ or return that there is no such hitting set. This approach has a major drawback as it does not give a solution for sets with a considerable hitting set. In this supplementary algorithm, we show that even with this major limitation, an exact algorithm that exhaustively searches for small Hitting set groups is inefficient and unscalable in real life, by giving a lower bound on the complexity of this approach and using a real-life example. This approach can be implemented using the following algorithm:

Sub Algorithm 0 Exact Algorithm for HS of size at most K (S, C, k)

*Input: universe* $S=\left\{ s_{1},\ldots, s_{m} \right\}, C=\left\{ C_{1}, \ldots, C_{n} \right\} s.t. C_{i}\subseteq S for all 1\leq i\leq n, constant k$

*0utput*: $\hat{S}\subseteq S s.t. \left| \hat{S} \right|\leq k$.

*1. for each* $\hat{S}\subseteq S$ *of size at most* $k$ *do*

*(a) Return* $\hat{S}$ *if for all* $C_{i}\subseteq C$*:* $\hat{S}\cap C_{i}\neq\emptyset$

*2.* $Return \emptyset$

Assuming we wish to find a solution of size at most $k$ for an instance of the problem with $m$ elements and $n$ subsets. An exhaustive search requires each possible set of size at most $k$ to be examined. Since each solution of size $k^{'}<k$ is a subset of larger hitting set of size $k$, we assume in this analysis that we can check for sets of exactly size $k$. There are $({m \atop k})$ such sets; for each one, we need to check whether it hits all $n$ subsets. We further leniently assume that checking if a potential HS hits a single input subset can be done in $O(1)$ on average with an appropriate data structure. Hence, the worst-case number of checks to find a hitting set of size $k$ is at least $n\cdot({m \atop k})$. For $k\ll m$ we have $n\cdot\binom{m}{k}\approx n\cdot\frac{m^{k}}{k!}$ by Stirling's approximation. As $n$, $m$ and $k$ grow, it becomes more and more impractical to search for a hitting set in this naive method.

For example, a feasible use for this Algorithm may result in the following parameters: $n=100, m=5000, k=5$. Thus, the number of check operations would be more than $\sim2.6e^{18}$ in the worst case, which will take years to compute on modern computers.

Table 1: hitting sets (marker proteins) of 17 microorganism groups. HS, hitting set. Min., minimal. Greedy and random refer to the algorithm type. Phen., phenotypic. Tax., taxonomic. AIEC, adherent-invasive *E. coli*. EPEC, enteropathogenic *E. coli*. UPEC, uropathogenic *E. coli*. STEC, Shiga toxin-producing *E. coli*. NMEC, neonatal meningitis-associated *E. coli*. ExPEC, extra-intestinal pathogenic *E. coli*. ETEC, enterotoxigenic *E. coli*. EIEC, enteroinvasive *E. coli*. EHEC, enterohemorrhagic *E. coli*. EAEC, enteroaggregative *E. coli*. APEC, avian pathogenic *E. coli*. EAHEC, enteroaggregative hemorrhagic *E. coli*.
